# High Genetic Diversity and *Rickettsia felis* in *Pediculus humanus* Lice Infesting Mbuti (pygmy people), -Democratic Republic of Congo

**DOI:** 10.3389/fcimb.2022.834388

**Published:** 2022-03-02

**Authors:** Nadia Amanzougaghene, Rezak Drali, Jean-Christophe Shako, Bernard Davoust, Florence Fenollar, Didier Raoult, Oleg Mediannikov

**Affiliations:** ^1^Aix Marseille Univ Institut de Recherche pour le Développement (IRD), Assistance Publique - Hôpitaux de Marseille (APHM), Microbes, Evolution (MEPHI), Phylogénie et Infection, Marseille, France; ^2^Institut Hospitalo-Universitaire (IHU)-Méditerranée Infection, Marseille, France; ^3^Plateforme Génomique - Bioinformatique, Institut Pasteur d’Algérie, Rue du Petit Staouéli, Algiers, Algeria; ^4^Plague Reference Laboratory, Bunia, Congo; ^5^Aix Marseille Univ, Institut de Recherche pour le Développement (IRD), Assistance Publique - Hôpitaux de Marseille (APHM), Vecteurs – Infections Tropicales et Méditeranéennes (VITROME), Marseille, France

**Keywords:** Mbuti pygmies, *Pediculus humanus*, genetic diversity, *Rickettsia felis*, Democratic Republic of Congo

## Abstract

*Pediculus humanus* is an obligate bloodsucking parasite of humans that has two ecotypes, the head louse and the body louse, which share an intimate history of coevolution with their human host. In the present work, we obtained and analysed head and body lice collected from Mbuti pygmies living in the Orientale province of the Democratic Republic of the Congo. *Cytochrome b* DNA analysis was performed in order to type the six known lice clades (A, D, B, F, C and E). The results revealed the presence of two mitochondrial clades. Clade D was the most frequent (61.7% of 47), followed by clade A (38.3% of 47). Sixteen haplotypes were found in 47 samples, of which thirteen were novel haplotypes, indicating an unusually high genetic diversity that closely mirrors the diversity of their hosts. Moreover, we report for the first time the presence of the DNA of *R. felis* in three (6.4% of 47) head and body lice belonging to both clades A and D. Additional studies are needed to clarify whether the *Pediculus* lice can indeed transmit this emerging zoonotic bacterium to their human hosts.

## Introduction

Human sucking lice from the genus *Pediculus* are obligate host-specific parasites, that co-evolved with their host (Reed et al., 2004; Amanzougaghene et al., 2020a). *P. humanus* includes two subspecies, each occupying a different ecological niche: hair for the head louse, *P. humanus capitis*, and garments for the body louse, *P. h. humanus* (Amanzougaghene et al., 2020a). Although morphologically almost identical, it is now possible to genetically differentiate the head louse from the body louse (Drali et al., 2013).

The body louse is the vector of three pathogenic bacteria that cause serious diseases, that killed millions of people, namely *Rickettsia prowazekii* (the causative agent of epidemic typhus), *Bartonella quintana* (trench fever) and *Borrelia recurrentis* (relapsing fever) (Raoult and Roux, 1999). In addition, body and head lice may harbour *Yersinia pestis*, the agent of plague (Houhamdi et al., 2006; Ayyadurai et al., 2010; Piarroux et al., 2013; Drali et al., 2015; Raoult, 2016). Moreover, experimental models showed that body lice have the potential to acquire, maintain and transmit *R. typhi* (the causative agent of endemic or murine typhus), *R. rickettsii* (Rocky Mountain spotted fever), *R. conorii* (Mediterranean spotted fever, Indian tick typhus), and *Acinetobacter baumannii* and *A. lwoffii* to rabbits (Weyer, 1952a; Houhamdi et al., 2003; Houhamdi and Raoult, 2006a; Houhamdi and Raoult, 2006b). *Coxiella burnetii*, the agent of Q fever, was first isolated from the body lice of individuals living in an epidemic area in Rwanda, and was subsequently found to infect body lice under experimental conditions (Giroud and Jadin, 1954; Babudieri, 1959). Recently, its DNA was found in body lice infecting homeless people in Algeria, as well as in body lice from France (Louni et al., 2018; Amanzougaghene et al., 2020b). The DNA of *Anaplasma phagocytophilum* was also detected in body lice from homeless people in Algeria (Louni et al., 2018). Although no reports in the field have yet demonstrated that head lice are vectors of infectious agents, several experimental and epidemiological reports indicate that they can transmit pathogens to their human host under favourable epidemiological conditions (Amanzougaghene et al., 2020a). Indeed, under laboratory conditions it has been shown that head lice retrieved from patients and rabbits infected with *R. prowazekii*, can be readily infected and disseminate this bacterium in their faeces, demonstrating that head lice have the potential to be a vector of this pathogen (Goldberger and Anderson, 1912; Murray and Torrey, 1975). Moreover, in a laboratory-reared head louse colony, it has been demonstrated that head lice can maintain a persistent *B. quintana* infection for several days following its acquisition in a bloodmeal (Previte et al., 2014). In addition, the DNA of several pathogenic bacteria have been reported in head lice, including *B. quintana, C. burnetii, B. recurrentis, Y. pestis*, *R*. *aeschlimannii, Anaplasma*, *Ehrlichia* and *Acinetobacter* spp. (Piarroux et al., 2013; Drali et al., 2015; Amanzougaghene et al., 2016; Amanzougaghene et al., 2017; Candy et al., 2018; Boumbanda Koyo et al., 2019; Amanzougaghene et al., 2020b; Boumbanda-Koyo et al., 2020; Hammoud et al., 2021).

**Figure 1:**
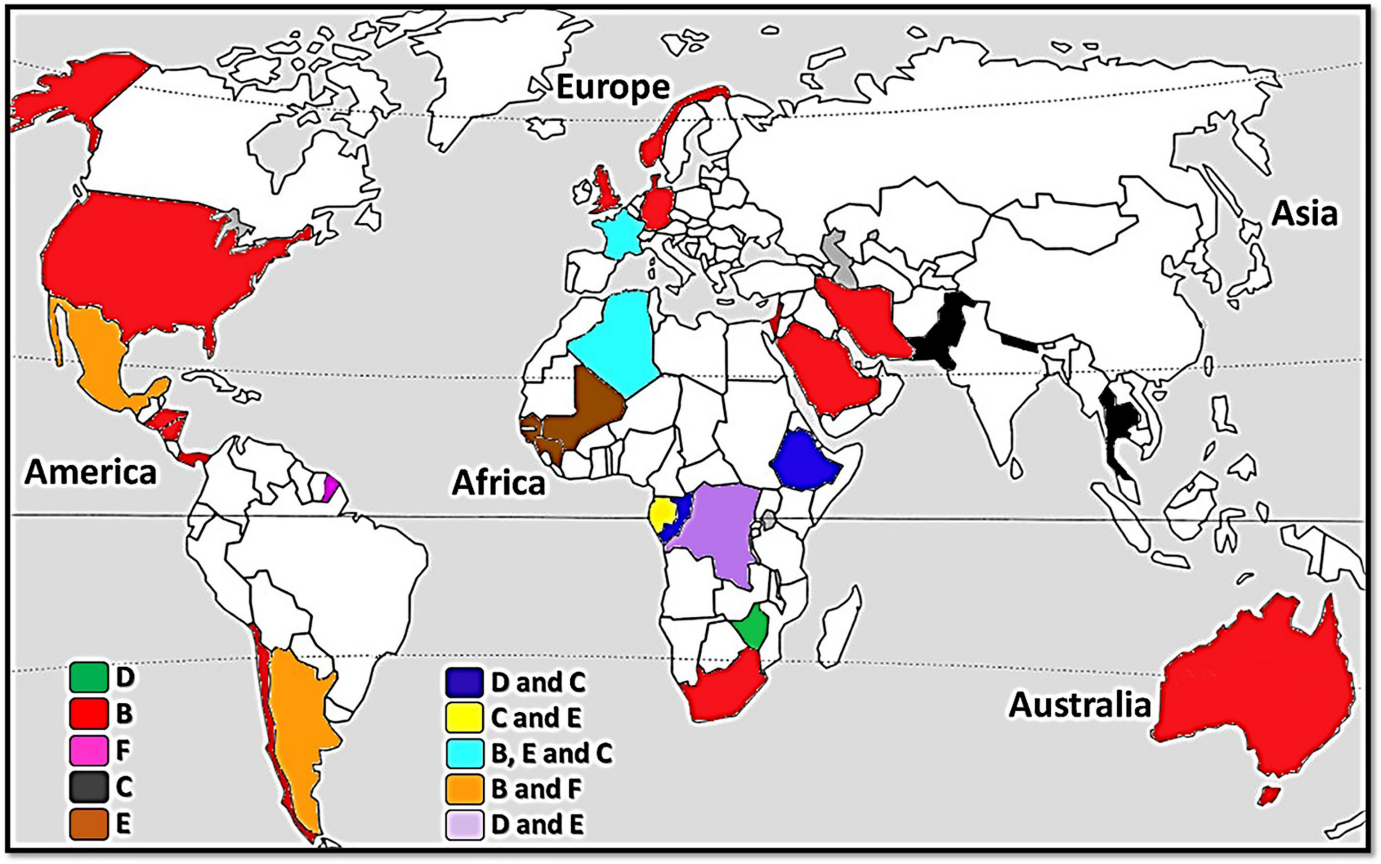
A map of the world showing the distribution of *Pediculus humanus* clades.

On the basis of all genetic analyses performed on human lice, only those targeting the mitochondrial genes were able to classify them into six distinctly different clades (Light et al., 2008; Ascunce et al., 2013; Ashfaq et al., 2015; Drali et al., 2015; Amanzougaghene et al., 2019). Head lice belong to all six clades (A, D, B, F, C and E), while body lice belong only to clades A and D (Amanzougaghene et al., 2019; Amanzougaghene et al., 2020a). Clade A is distributed worldwide (Ascunce et al., 2013; Ashfaq et al., 2015; Amanzougaghene et al., 2019), whereas the other clades are geographically restricted (**Figure 1**). Clade D is restricted to sub-Saharan African countries, including the Democratic Republic of Congo (DRC) and the Republic of Congo (Congo-Brazzaville), Ethiopia and Zimbabwe (Drali et al., 2015; Amanzougaghene et al., 2019). Clade B includes head lice found in the Americas, Western Europe, Australia, North and South Africa, Saudi Arabia and Iran (Light et al., 2008; Ascunce et al., 2013; Ashfaq et al., 2015; Boutellis et al., 2015; Al-Shahrani et al., 2017). Clade F, which is the sister group of clade B, is found in South America (Amanzougaghene et al., 2019; Amanzougaghene et al., 2020a). Clade C is, to date, limited to Africa and Asia (Ashfaq et al., 2015; Sunantaraporn et al., 2015; Amanzougaghene et al., 2016; Boumbanda-Koyo et al., 2020). Finally, clade E has mainly been found in West African countries (Senegal, Mali and Guinea) (Amanzougaghene et al., 2017; Amanzougaghene et al., 2020a; Hammoud et al., 2021).

In addition to their inter clade diversity, human lice also display high intra clade diversity, as illustrated by the multiple distinct haplotypes for each clade (Ascunce et al., 2013; Ashfaq et al., 2015; Amanzougaghene et al., 2016; Drali et al., 2016; Amanzougaghene et al., 2019). The genetic diversity is also observed among global populations of *Homo sapiens*. It is even more accentuated among the indigenous populations of Africa, especially among pygmies. Indeed, the genetic diversity found among African pygmies is the most significant in the world. They present great diversity, both among themselves and compared to other African populations (Henn et al., 2011; Duda and Jan Zrzavý, 2016). The group is characterised by their reduced height and they survive from hunting and gathering in the rain forest in Central Africa (Jakobsson et al., 2008; Campbell and Tishkoff, 2010; Batini et al., 2011). Their number varies between 300,000 and 500,000 individuals distributed into 25 ethno-linguistic groups (Ohenjo et al., 2006; Becker et al., 2011). Molecular dating analysis based on mitochondrial DNA has revealed that they shared a common ancestor with non-pigmy populations dating to around 60 thousand years ago, whereas the separation between the West and East African pygmies dates to approximately 20 thousand years ago (Destro-Bisol et al., 2004; Batini et al., 2011; Jarvis et al., 2012). African pygmies are known to be at the root of the modern human tree (Jakobsson et al., 2008). Archaic DNA was recently characterised in the Mbuti pygmies from the Itury forest in the Republic Democratic of the Congo (RDC) (Hammer et al., 2011). Mbuti pygmies are relatively isolated from other Pygmy and neighbouring non-Pygmy populations and are directly descended from the ancestors of most Pygmy groups (Patin et al., 2009; Verdu and Destro-Bisol, 2012).

In this study, we were fortunate to obtain and analyse head and body lice collected from pygmy populations that belong to the Mbuti ethnic group living in the Orientale province of the Democratic Republic of the Congo, where the Clade D of human lice was characterised for the first time (Drali et al., 2015).

## Materials and Methods

### Ethics Statement

This study was approved by the by the Ethics Committee of the École de Santé Publique de l’Université de Kinshasa (N/Réf:ESP/CE/17B/2014). The head and body lice were collected from infested individuals after obtaining their verbal consent. Approval was obtained from the local authorities and representatives were present when it was performed. Moreover, the collection of lice from humans is a non-invasive procedure. This it was made according to the prescriptions of the Declaration of Helsinki of the World Medical Association.

### Louse Sampling

**Figure 2:**
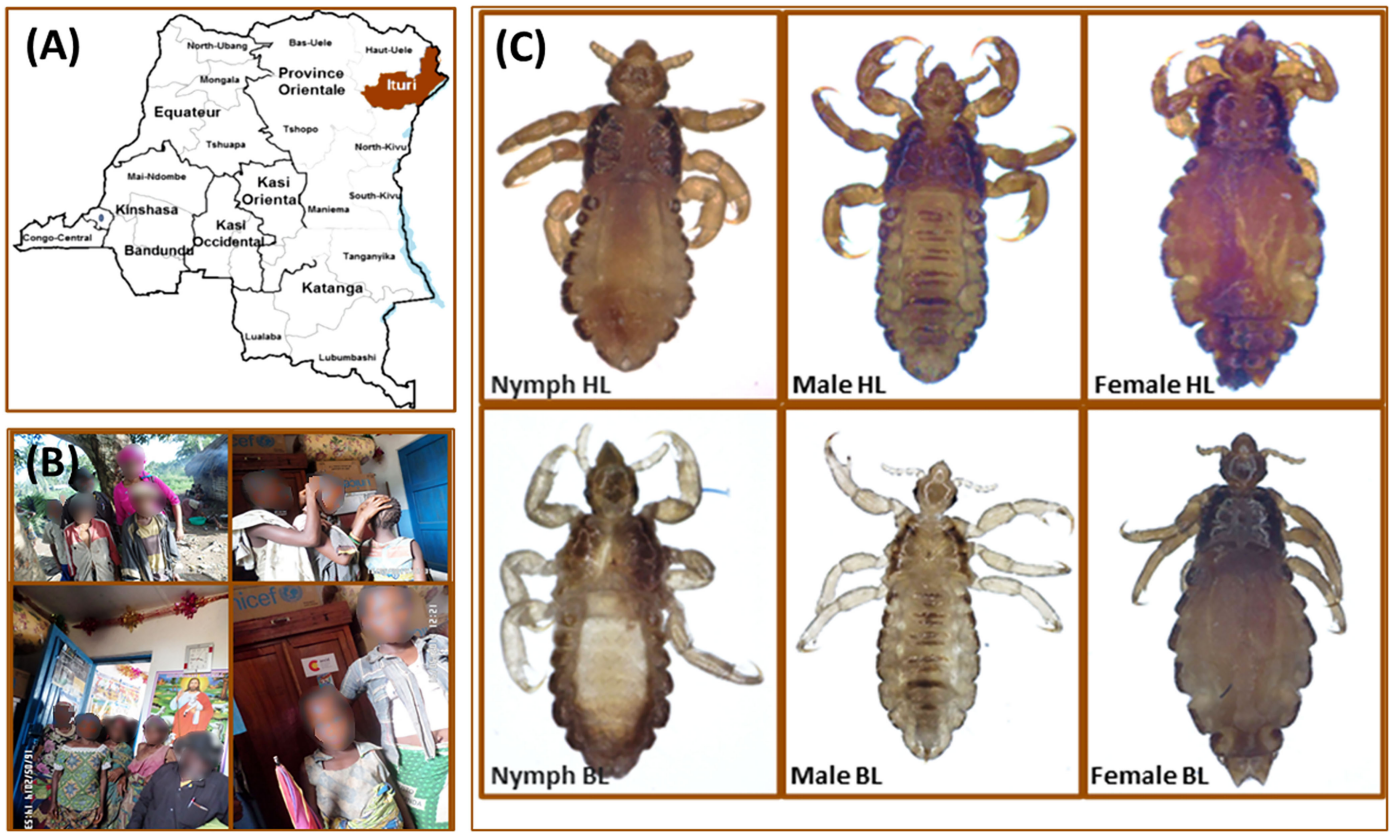
Head and body lice collected from Mbuti pygmies, living in the Orientale province of the Democratic Republic of the Congo. **(A)** Geographical location of louse sampling. **(B)** Mbuti pygmies infested with lice. **(C)** Head and body lice from Mbuti pygmies.

This study was carried out in March 2014 on Mbuti Pygmies living in Shaurimoya (1°40′14″ N, 28°29′21″ E), a remote rural village, located in the Itury rain forest area near the Rethy Health District, in the Orientale province of the Democratic Republic of the Congo (**Figures 2A, B**). A total of 47 lice (22 body lice and 25 head lice) were collected from 22 infested volunteers aged between 4 and 30 years old. All head lice were collected exclusively from the hair, and all body lice were collected exclusively from clothing. The collected lice were then preserved in 70% ethanol before being sent to our laboratory in Marseille (France). Once received at the laboratory, the specimens were photographed on their dorsal and ventral sides using a fixed camera (Olympus DP71, Rungis, France).

### DNA Extraction

Total DNA was extracted from lice using a DNA extraction kit, the QIAamp Tissue Kit (Qiagen SAS, Courtaboeuf, France) with the EZ1 apparatus following the manufacturer’s protocol. DNA from each louse was eluted in 100 μl of TE buffer and stored at -20°C under sterile conditions until the next stage of the investigation.

### Lice Clade and Phylogenetic Analysis

For phylogenetic analysis, the cytochrome b *(cytb)* gene was chosen, as it was the most commonly used in human louse phylogenetic studies (Li et al., 2010; Ashfaq et al., 2015; Amanzougaghene et al., 2016; Amanzougaghene et al., 2019). A polymerase chain reaction (PCR) was performed to amplify a fragment of 347-bp of *cytb* gene using the primers described previously (Li et al., 2010), in a MiniAmp™ Plus Thermal Cycler (Thermo Fisher Scientific, Illkirch, France). PCRs consisted of 50 µl volume including 25 µl Amplitaq gold master mix (Applied Biosystems, Foster City, CA, USA), 1 µl of each primer, 5 μl of DNA template, and water. The thermal cycling profile was one incubation step at 95°C for 15 minutes, 40 cycles of one minute at 95°C, 30 seconds at 56°C and one minute at 72°C followed by a final extension for five minutes at 72°C. Negative and positive controls were included in each assay. PCR products were purified using NucleoFast 96 PCR plates (Macherey-Nagel EURL, Hoerdt, France) as per the manufacturer’s instructions. The amplicons were sequenced using the Big Dye Terminator Cycle Sequencing Kit (Perkin Elmer Applied Biosystems, Foster City, CA) with an ABI automated sequencer (Applied Biosystems). The obtained electropherograms were assembled and edited using ChromasPro software (ChromasPro 1.7, Technelysium Pty Ltd., Tewantin, Australia) and compared with those available in the GenBank database by NCBI BLAST (http://blast.ncbi.nlm.nih.gov/Blast.cgi). For all *cytb* nucleotides sequences obtained in this study, unique haplotypes were defined using DnaSPv5.10 and compared with the most recent *cytb* haplotype database described previously (**Supplementary Table S1**). Moreover, to learn more about the diversity and the genetic relatedness of lice infesting different populations of African pygmies, we recovered and analysed 160 *cytb* sequences from the pygmy lice of Congo Brazzaville (Amanzougaghene et al., 2016), that we compared to those included in this study. All the pygmies’ haplotypes, together with the references from all the body and head lice clades were used to construct maximum-likelihood (ML) trees and a median-joining (MJ) network. To generate the best ML tree, the Modeltest v.3.7 (Posada and Crandall, 1998) was used to examine model of nucleotide substitution and choose a best-fit model of sequence evolution. The HKY+I+G model was chosen as the best model of evolution, providing the best approximation of the data using the fewest parameters according to the Akaike Information Criterion (Huelsenbeck and Rannala, 1997; Posada and Buckley, 2004). Trees reconstructions were conducted using MEGA 6 software (Tamura et al., 2013) with ML method under HKY + I + G model with 500 bootstrap replicates. *Cytb* sequence from *P. schaeffi* (accession number: AY696067) was employed as outgroup. The median-joining (MJ) network was constructed using the Bandelt method with the NETWORK5.0 programme (www.fluxus-engineering.com/sharenet.htm) using equal weights for all mutations (Bandelt et al., 1999). For genetic diversity and haplotype analysis, population genetic indices including number of sequences (n), number of polymorphic sites (S), average number of pairwise nucleotide differences (k), nucleotide diversity (π), number of haplotypes (H) and haplotype diversity (Hd) and neutrality tests (Fu & Li’s D and Tajima’s D) were calculated for all *cytb* sequences, using DNASP v5.10 software (Librado and Rozas, 2009). The newly generated cytb sequences identified were submitted to the GenBank database under the accession numbers: MH117941-MH117953.

### Molecular Detection of the Presence Of Pathogen DNA

Real-time quantitative PCR (qPCR) was performed to test each DNA sample for the presence of *R. prowazekii*, *Rickettsia* spp., *Y. pestis*, *B. quintana*, *Borrelia* spp., *C. burnetii Anaplasma* spp., Piroplasmida (*Theileria* spp. and *Babesia* spp.) and *R. felis* using previously reported specific primers and probes. All *R. felis* positive samples were confirmed by a second specific qPCR targeting the *Orfb* gene. All sequences of primers and probes used for qPCRs in this study are shown in **Supplementary Table S2**. All qPCRs were performed using a CFX96 Real-Time system (Bio-Rad, Marnes-la-Coquette, France) and the Roche LightCycler 480 Probes Master Mix PCR kit (Roche Applied Science, Mannheim, Germany) in accordance with the manufacturer’s instructions. We included the DNA of the target bacteria as positive controls and master mixtures as a negative control for each qPCR run.

## Results

**Figure 3:**
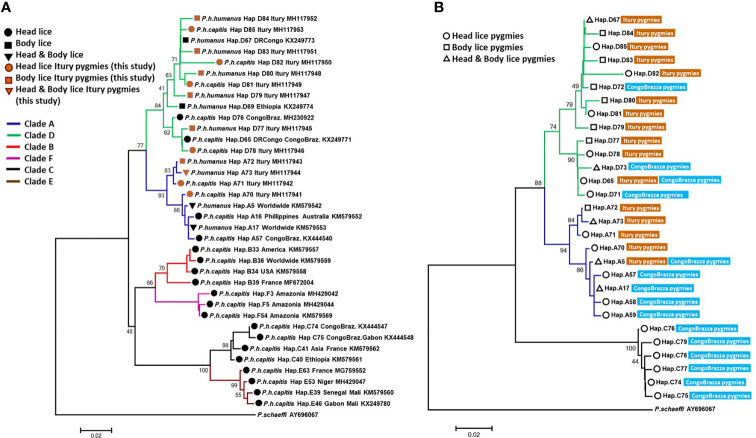
The phylogeny of head and body lice from Mbuti pygmies based on the *cytb* gene. Maximum-likelihood phylogenetic tree showing the relationship of haplotypes identified in this study **(A)** with the most prevalent haplotypes of *P. humanus* and **(B)** haplotypes from head lice Congo Brazzaville pygmies. Phylogenetic inference was conducted in MEGA 7 using the maximum likelihood method under HKY + I + G model with 500 bootstrap replicates. There was a total of 270 positions in the final dataset. The scale-bar represents a 2% nucleotide sequence divergence.

**Figure 4:**
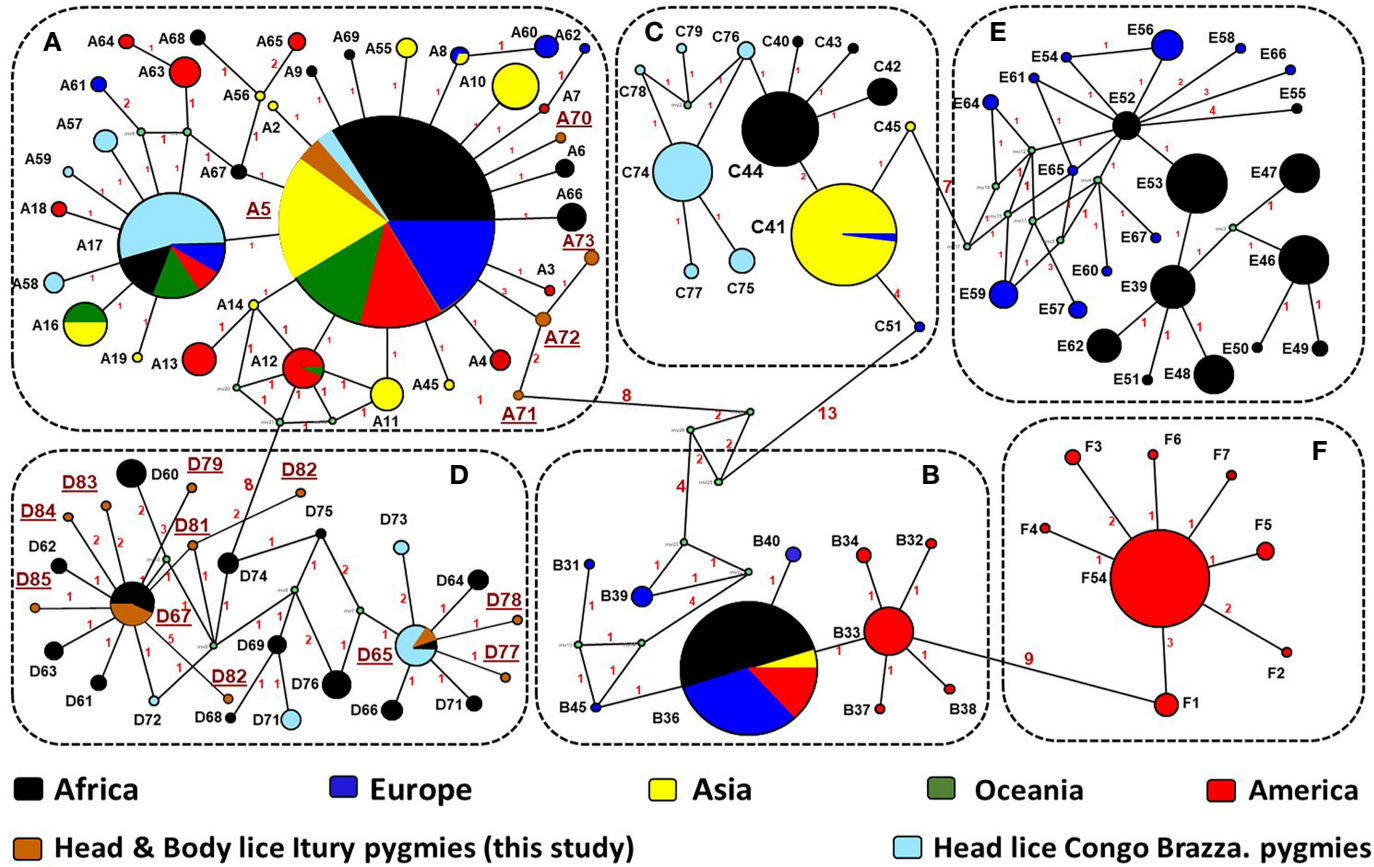
*Cytb* haplotype networks of *Pediculus* body and head lice. Each circle indicates a unique haplotype (272 bp) and variations in circle size are proportional to haplotype frequencies. Pie colours and sizes in circles represent the continents and the number of their sequence for a haplotype. The length of the links between nodes is proportional to the number of mutations. The types of haplotypes identified in this study are underlined.

The microscopic examination of morphological criteria such as size, shape and color of the lice recovered from pygmies analyzed in this study revealed no morphological discordance in relation to what is common in human lice genus *Pediculus* (**Figure 2C**). The mitochondrial DNA analysis, of the 47 specimens (26 head lice and 21 body lice) collected from 22 mono and/or double infested individuals, showed a distribution of 38.3% for haplogroup A (12 head and 17 body lice) and 61.7% for haplogroup D (12 head and 17 body lice). Among the 22 individuals, eight (21.6%) showed dual infestation with both clades and three (8%) were simultaneously infested with both body and head lice (**Table 1**). An in-depth analysis of all obtained nucleotide sequences and their alignment with all publicly available haplotypes revealed the presence of 16 haplotypes in 47 samples, defined by variation of 30 nucleotide positions (18 transitions and 12 transversions) (**Table 2**), indicating an unusually high genetic diversity among the lice studied from Mbuti pygmies. One haplotype (12 nucleotide sequences) belonged to haplotype A5 within clade A, which is the most widely distributed haplotype in the world. Two haplotypes within clade D, haplotype D65 (seven sequences) and D67 (13 sequences), which are the most prevalent haplotypes among clade D, were also shared with pygmy lice from Congo-Brazzaville (**Figures 3A, B** and **4**). Lastly, the remaining 13 haplotypes, A70-A71 (one sequence for each haplotype) and A72-A73 (two sequences for each haplotype), all belonged to clade A, and D77-D85 (one sequence for each haplotype) belonged to clade D, were novel and unique to lice from Mbuti pygmies (**Figure 4**).

**Table 1:** Summary of the pathogens detected in body and head lice collected from infested Mbuti pygmies in Itury, Congo-DRC.

Patients	Type of lice		N	Clade A	Clade D	Detection of
	Head (N)	Body (N)				*R. felis*
**Patient 1**	3	2	**5**	4	1	–
**Patient 2**	1		**1**	1		–
**Patient 3**	1		**1**		1	1 (BL)
**Patient 4**		1	**1**		1	–
**Patient 5**	1	5	**6**	1	5	–
**Patient 6**		5	**5**	1	4	–
**Patient 7**		3	**3**	1	2	–
**Patient 8**		2	**2**	1	1	–
**Patient 9**	2		**2**		2	–
**Patient 10**	1		**1**		1	–
**Patient 11**	1		**1**	1		–
**Patient 12**	2		**2**	1	1	–
**Patient 13**	1		**1**		1	–
**Patient 14**	4		**4**	2	2	–
**Patient 15**	3		**3**	2	1	–
**Patient 16**		2	**2**		2	–
**Patient 17**	1		**1**		1	–
**Patient 18**	1		**1**	1		–
**Patient 19**	1	1	**2**	1	1	2 (HL and BL)
**Patient 20**		1	**1**	1		–
**Patient 21**	1		**1**		1	–
**Patient 22**	1		**1**		1	–
***Total***	25	22	47	18	29	3

N, number; BL, Body louse; HL, Head louse.

**Table 2:** Haplotype frequency of head and body lice identified from infested pygmy individuals in Itury, Congo-RDC.

Clade of lice	Haplotypes	Number	H or B	GenBank ID	Amplicon size
**Clade A**	A5	12	H B	KM579542	272 bp
	A70	1	H	MH117941	272 bp
	A71	1	H	MH117942	272 bp
	A72	2	B	MH117943	272 bp
	A73	2	H B	MH117944	272 bp
**Clade D**	D65	7	H	KX249771	272 bp
	D67	13	H B	KX249773	272 bp
	D77	1	B	MH117945	272 bp
	D78	1	H	MH117946	272 bp
	D79	1	B	MH117947	272 bp
	D80	1	B	MH117948	272 bp
	D81	1	H	MH117949	272 bp
	D82	1	H	MH117950	272 bp
	D83	1	B	MH117951	272 bp
	D84	1	B	MH117952	272 bp
	D85	1	H	MH117953	272 bp
**Total**	**16 haplotypes**	**47 Lice**	**26 H and 21 B**		

The new haplotypes identified in this study are underlined.

From the 160 *cytb* sequences of the pygmy lice from Congo Brazzaville (Amanzougaghene et al., 2016) that we analysed, 15 haplotypes were identified (**Table 3**). Among them only two haplotypes (A5 and D65) were shared with those of Mbuti pygmies, as it was shown in the **Figure 3B**. These 15 haplotypes are well distributed across three clades, A, D and C, while those of Mbuti pygmy lice belonged only to clades A and D (**Figure 3B**). The MJ network analysis for all *cytb* haplotypes corroborated the ML phylogenetic reconstruction 3, 4), with all the *cytb* sequences were divided across separate clusters represented by six connected subnetworks corresponding to the six know clades A, D, B, C, E and F. Estimates of genetic diversity indices and the results of neutrality tests for all *cytb* sequences are presented in **Table 3**. The average number of nucleotide diversity (π), pairwise nucleotide differences (k) and haplotype diversity (Hd) varied among the six clades and among *cytb* sequences of pygmy lice from Mbuti and Congo Brazzaville. The highest haplotype diversity was found within Mbuti pygmies (Hd= 0.846) when compared to those of Congo Brazzaville pygmies (Hd= 0,773). Within the different clades, the highest haplotype diversity was found in clade D (Hd=0.831), which mainly includes the haplotypes identified in lice from Mbuti and Congo-Brazzaville pygmies (**Table 3**).

**Table 3:** Analysis of genetic diversity indices and neutrality tests (Fu & Li’s D and Tajima’s D) on mitochondrial *cytb* sequences.

	n	S	K	π	h	Hd	Fu & Li’s D	Tajima’s D
Cytb all	1500	96	20.341	0.075	105	0.999	-2,878 (P <0.05) S*	-0.172 (P> 0.1) NS
Clade A	769	33	3.098	0.011	34	0.750	-3.751 (P <0.02) S**	-2.322 (P < 0.01) S**
Clade D	69	20	5.044	0.018	17	0.831	-1.425 (P> 0.1) NS	-0.583 (P> 0.1) NS
Clade B	200	13	3.694	0.013	9	0.789	-1.633 (P> 0.1) NS	-1.357 (P> 0.1) NS
Clade F	104	11	2.750	0.010	9	0.700	-1.924 (P < 0.05) S*	-1.757 (P < 0.05) S*
Clade C	205	16	3.744	0.014	13	0.792	-1.471 (P> 0.1) NS	-1.151 (P> 0.1) NS
Clade E	153	26	4.075	0.015	23	0.803	-2.747 (P < 0.05) S*	-1.752 (P > 0.05) NS
*Cytb* Mbuti	47	30	8,551	0,0314	16	0,846	-1,3904 (P > 0.1) NS	0,6086 (P> 0.1) NS
*Cytb* Braz.	160	46	13,905	0,0511	15	0,773	0,3687 (P > 0.1) NS	2,0265 (P > 0.1) NS

n, number of sequences; S, number of polymorphic sites; k, average number of pairwise nucleotide differences; π, nucleotide diversity; h, number of haplotypes; Hd, haplotype diversity. Tajima’s D: A negative Tajima’s D signifies an excess of low frequency polymorphisms relative to expectation. A positive Tajima’s D signifies low levels of both low and high frequency polymorphisms. Statistical significance: Not significant, P > 0.10. Braz., Brazzaville. The new haplotypes identified in this study are underlined. NS, not significant; *P < 0.05; **P < 0.01.

In this study we also screened all louse samples for the presence of potential louse-borne pathogens. The results showed that all qPCR investigation of 47 head and body louse samples for *Borrelia* spp., *Rickettsia* spp., *Anaplasma* spp., *R. prowazekii*, *B. quintana*, *Y. pestis*, *C. burnetii* and Piroplasmida (*Theileria* spp. and *Babesia* spp.) spp. produced no positive results. However, we identified *R. felis* in three lice specimens. The DNA of *R. felis* (two independent specific qPCRs targeting different genes) was detected in three of 47 (6.4%) louse specimens recovered from two individuals (9.1% of 22) (Patient 3 and Patient 19, see **Table 1**). Two positive lice were collected from the same patient (Patient 19): one head louse belonging to clade A and one body louse from clade D. The third positive louse also belonged to clade D and was recovered from another patient (Patient 3).

## Discussion

To the best of our knowledge, this study reports for the first time molecular data for human lice, genus *Pediculus*, infesting Mbuti Pygmies living in the Democratic Republic of the Congo (**Figure 2**). The mtDNA analysis revealed that the 47 lice from Mbuti Pygmy individuals investigated in this work belong to two different *cytb* haplogroups, A and D, distributed through 16 haplotypes. Clades A and D have already been reported in this country and constitute the two dominant louse lineages in this area (Drali et al., 2015; Boumbanda Koyo et al., 2019). Clade A is globally distributed, while Clade D, has thus far only been found in Central African countries including DR Congo and Congo-Brazzaville, Ethiopia, and Zimbabwe (Amanzougaghene et al., 2016; Amanzougaghene et al., 2019). Clade D is the sister group of clade A from which is thought to have diverged between 0.37 and 0.54 million years ago (Ashfaq et al., 2015; Amanzougaghene et al., 2019).

Although this study includes only a small number of lice (47) and 22 humans. This is mainly due to the fact that these populations of pygmies live in small groups (the average size of a group in Mbuti varies between 15 to 60 individuals), in remote areas of the forest which are very difficult to access. However, it is possible to talk of great genetic variability among these lice. This result is supported by our finding, that, of the 47 Mbuti lice *cytb* sequences analyzed, sixteen haplotypes were found, of which thirteen were novel haplotypes. Moreover, we were already aware of the intimate history of the coevolution between lice and their human host and so were not surprised by the results of this study. While we did not have the opportunity to carry out genetic analyses of the carriers of these 47 lice, the literature shows that the genetic variability among pygmies is certainly the greatest in the world. They are significantly differentiated, both among themselves and compared to other African populations (Henn et al., 2011; Duda and Jan Zrzavý, 2016). This is especially true, since this study targeted Mbuti pygmies, who are known to be relatively isolated from other pygmy and neighbouring non-pygmy populations (Patin et al., 2009; Verdu and Destro-Bisol, 2012). The results obtained from the phylogenetic analysis of lice from pygmy people from the Republic of the Congo (Congo Brazzaville) have also shown a certain genetic diversity, albeit less pronounced than that observed in Mbuti lice (Amanzougaghene et al., 2016). They are well distributed across two clades, A and C, the two most well distributed clades in Africa (Amanzougaghene et al., 2016). Moreover, as is the case of lice from Itury individuals, they are absent from clades B, E and F but share clade D with them. Of the 11 haplotypes present, eight are specific to them (**Figures 3B** and **4**) (Amanzougaghene et al., 2016). This supports the statement that pygmies in Africa have exceptional genetic diversity (Henn et al., 2011; Duda and Jan Zrzavý, 2016).

*Rickettsia felis*, the causative agent of flea-borne spotted fever, is a worldwide emerging zoonosis and an important cause of human febrile illness in Africa, including the DR Congo (Mediannikov et al., 2012; Angelakis et al., 2016; Legendre and Macaluso, 2017). The cat flea, *Ctenocephalides felis*, was considered to be the only confirmed biological vector of *R. felis* (Angelakis et al., 2016). However, various other arthropods including fleas, mosquitoes, ticks, mites, lice and bed bugs have been found to harbour *R. felis* (see **Supplementary Table S3**) (Mediannikov et al., 2014). Moreover, *Anopheles gambiae* was recently proposed as a potential vector of *R. felis* (Dieme et al., 2015). In the DR Congo, this bacterium was frequently detected from several arthropods, including *C. felis*, *C. canis*, *Pulex irritans*, *Echidnophaga gallinacea*, *Xenopsylla brasiliensis*, *Tunga penetrans* and *Leptopsylla aethiopica* (**Supplementary Table S3**) (Sackal et al., 2008; Mediannikov et al., 2012; Leulmi et al., 2014). In this study, we report for the first time, to the best of our knowledge, the presence of *R*. *felis*-DNA in three lice (one head and two body) recovered from two Mbuti pygmies. Although *R*. *prowazekii* is the only *Rickettsia* species known to be naturally associated with human lice, several experimental studies have demonstrated that the body louse may play a role, under favourable epidemiologic circumstances, in the transmission of other *Rickettsia* species to humans (Raoult and Roux, 1999; Amanzougaghene et al., 2020a). This is the case of *R. akari* (rickettsial pox) and *R. typhi* (endemic or murine typhus), which are typically transmitted, respectively, by acari mites and insect fleas, *R. conorii* (Mediterranean spotted fever, Indian tick typhus) and *R. rickettsii* (Rocky Mountain spotted fever), both of which are transmitted by ticks (Weyer, 1952b; Houhamdi et al., 2003; Houhamdi and Raoult, 2006b). Moreover, *R. typhi* has been isolated from body lice infesting sick patients during an outbreak of murine typhus that occurred in northern China and India (Kashmir State) (Liu, 1944; Kalra and Rao, 1951). Furthermore, in the epidemiological study conducted on Malian head lice, the DNA of another *Rickettsia*, namely *R*. *aeschlimannii*, was also detected in 2.5% of 600 head lice collected from 9.4% of 117 tested individuals (Amanzougaghene et al., 2017). Our results from the lice recovered from Mbuti pygmies, together with data from the literature, suggest that the role of human lice in the epidemiology of *R. felis* should be investigated further.

## Conclusion

In conclusion, our finding confirms the presence of clades A and D in body and head lice infesting Mbuti Pygmies living in the Democratic Republic of the Congo. Sixteen haplotypes were found in 47 samples, of which thirteen haplotypes were novel and unique to lice from Mbuti pygmies, indicating an unusually high genetic diversity and reflecting the diversity of their pygmy hosts. Interestingly, we report for the first time the presence of *R. felis*-DNA from both body and head lice belonged to both clades A and D, which suggest that *Pediculus* lice can act as a potential vector of this *Rickettsia* species.

## Data Availability Statement

The datasets presented in this study can be found in online repositories. The names of the repository/repositories and accession number(s) can be found in the article/**Supplementary Material**.

## Author Contributions

DR, OM, and RD conceived and designed the experiments. BD and J-CS collected samples. NA conducted the experiments. NA and RD analysed the data. NA, RD, BD, OM, and DR wrote the paper. All authors contributed to manuscript revision, read, and approved the submitted version.

## Funding

This study was supported by the Institut Hospitalo-Universitaire (IHU) Méditerranée Infection, the National Research Agency under the “Investissements d’avenir” programme, reference ANR-10-IAHU-03, the Région Provence-Alpes-Côte d’Azur and European ERDF PRIMI funding.

## Conflict of Interest

The authors declare that the research was conducted in the absence of any commercial or financial relationships that could be construed as a potential conflict of interest.

## Publisher’s Note

All claims expressed in this article are solely those of the authors and do not necessarily represent those of their affiliated organizations, or those of the publisher, the editors and the reviewers. Any product that may be evaluated in this article, or claim that may be made by its manufacturer, is not guaranteed or endorsed by the publisher.
