# Supplementary material for: High Genetic Diversity and Rickettsia felis in Pediculus humanus Lice Infesting Mbuti (pygmy people), -Democratic Republic of Congo
Source: Front Cell Infect Microbiol. 2022 Mar 2;12:834388. doi: 10.3389/fcimb.2022.834388 (PMC8924665; doi:10.3389/fcimb.2022.834388)
Supplement: Supplementary file 1 [file DataSheet_1.docx]

**Supplementary Materials**

**Supplementary Table S1.** Geographic occurrences and frequencies of *cytb* haplotypes of human head and body lice.

**Supplementary Table S2.** Oligonucleotide sequences of primers and probes used for real-time PCRs and conventional PCRs in this study.

| **Target** | **Name** | **Primers (5’-3’) and probes** | **Source** |
| --- | --- | --- | --- |
| ***P. humanus***  *cytochrome b* | Duplex A-D | F_GATGTAAATAGAGGGTGGTT | (Amanzougaghene et al., 2017) |
|  |  | R_GAAATTCCTGAAAATCAAAC |  |
|  |  | FAM-CATTCTTGTCTACGTTCATATTTGG-TAMRA |  |
|  |  | VIC-TATTCTTGTCTACGTTCATGTTTGA-TAMRA |  |
|  | Duplex B-C/E | F_TTAGAGCGMTTRTTTACCC |  |
|  |  | R_AYAAACACACAAAAMCTCCT |  |
|  |  | FAM-GAGCTGGATAGTGATAAGGTTTAT-MGB |  |
|  |  | VIC-CTTGCCGTTTATTTTGTTGGGGTTT-TAMRA |  |
|  | Monoplex E | GGTTGGAATTGGATAGTGAT | (Amanzougaghene et al., 2017) |
|  |  | GGGTCCATAAAGAAATCC G |  |
|  |  | FAM- TAGGAGGCTTTGTGTGTCTATCCT-TAMRA |  |
|  | *Cytb* | F_GAGCGACTGTAATTACTAATC | (Li et al., 2010) |
|  |  | R_CAACAAAATTATCCGGGTCC |  |
| ***Acinetobacter* spp*.***  RNA polymerase β subunit gene | *rpoB* | F_TACTCATATACCGAAAAGAAACGG | (Bouvresse et al., 2011) |
|  |  | R_GGYTTACCAAGRCTATACTCAAC |  |
|  |  | FAM-CGCGAAGATATCGGTCTSCAAGC-TAMRA |  |
|  | *rpoB* (zone1) | F_TAYCGYAAAGAYTTGAAAGAAG | (La Scola et al., 2006) |
|  |  | R_CMACACCYTTGTTMCCRTGA |  |
| ***R. prowazekii***  *rOmpB* gene | *ompB* | F_AATGCTCTTGCAGCTGGTTCT | (Nguyen-Hieu et al., 2010) |
|  |  | R_TCGAGTGCTAATATTTTTGAAGCA |  |
|  |  | FAM-CGGTGGTGTTAATGCTGCGTTACAACA-TAMRA |  |
| ***Y. pestis***  plasminogen activator gene | PLA | F_ATGGAGCTTATACCGGAAAC | (Nguyen-Hieu et al., 2010) |
|  |  | R_GCGATACTGGCCTGCAAG |  |
|  |  | FAM-TCCCGAAAGGAGTGCGGGTAATAGG-TAMRA |  |
| ***Borrelia* spp.**  *16S ribosomal RNA* | Bor16S | F_AGCCTTTAAAGCTTCGCTTGTAG | (Parola et al., 2011) |
|  |  | R_GCCTCCCGTAGGAGTCTGG |  |
|  |  | FAM-CCGGCCTGAGAGGGTGAACGG-TAMRA |  |
| ***B. quintana***  Hypothetical intracellular effector | yopP | F_TAAACCTCGGGGGAAGCAGA | (Angelakis et al., 2011) |
|  |  | R_TTTCGTCCTCAACCCCATCA |  |
|  |  | FAM-CGTTGCCGACAAGACGTCCTTG-TAMRA |  |
| 3-oxoacyl-synthase gene | fabF3 | F_GCGGCCTTGCTCTTGATGA |  |
|  |  | R_GCTACTCTGCGTGCCTTGGA |  |
|  |  | FAM-TGCAGCAGGTGGAGAGAACGTG-TAMRA |  |
| ***Anaplasma* spp.**  23S ribosomal RNA | TtAna | F_TGACAGCGTACCTTTTGCAT | (Dahmani et al., 2015) |
|  |  | R_TGGAGGACCGAACCTGTTAC |  |
|  |  | FAM-GGATTAGACCCGAAACCAAG-TAMRA |  |
| ***C. burnetii***  Spacers IS1111 | IS1111 | F_CAAGAAACGTATCGCTGTGGC | (Mediannikov et al., 2010) |
|  |  | R_CACAGAGCCACCGTATGAATC |  |
|  |  | FAM_CCGAGTTCGAAACAATGAGGGCTG-TAMRA |  |
| ***Rickettsia* spp.**  Citrate synthase (*gltA*) | RKND03 | F_GTGAATGAAAGATTACACTATTTAT | (Sokhna et al., 2013) |
|  |  | R_GTATCTTAGCAATCATTCTAATAGC |  |
|  |  | FAM_CTATTATGCTTGCGGCTGTCGGTTC-TAMRA |  |
| ***R. felis***  Biotin synthase | 527 | F_ATGTTCGGGCTTCCGGTATG | (Socolovschi et al., 2010) |
|  |  | R_CCGATTCAGCAGGTTCTTCAA |  |
|  |  | FAM_GCTGCGGCGGTATTTTAGGAATGGG_TAMRA |  |
| ***R. felis***  Transposition helper protein | *Orfb* | F_CCCTTTTCGTAACGCTTTGCT | (Socolovschi et al., 2012b) |
|  |  | R_GGGCTAAACCAGGGAAACCT |  |
|  |  | FAM_TGTTCCGGTTTTAACGGCAGATACCCA_TAMRA |  |
| ***Piroplasmida* spp**  5.8S rRNA | 5.8S | F_AYYKTYAGCGRTGGATGTC | (Dahmana et al., 2019) |
|  |  | R_TCGCAGRAGTCTKCAAGTC |  |
|  |  | FAM-TTYGCTGCGTCCTTCATCGTTGT-MGB |  |

**Supplementary Table S3.** Detection of *R. felis* in various arthropod species

| **Group** | **Species** | **Country** | **Reference** |
| --- | --- | --- | --- |
| **Fleas** | *Ctenocephalides felis* | Albania, Australia, Brazil, China, Colombia, Costa Rica, **Democratic Republic of Congo**, Ethiopia, Guatemala, Hungary, Italy, Kenya, Laos, Lebanon, Malaysia, Mexico, New Caledonia, Netherlands, Panama, Peru, Spain, Taiwan, Tunisia, United Republic of Tanzania, USA, Uruguay, West Indies, Democratic Republic of Sao Tome and Principe, UK, France, Gabon | (Rolain et al., 2003; Venzal et al., 2006; Gehrke et al., 2009; Karpathy et al., 2009; Varagnol et al., 2009; Hornok et al., 2010; Kelly et al., 2010; Bermúdez et al., 2011; Hun et al., 2011; Mba et al., 2011; Mediannikov et al., 2011, 2012a, 2012b; Tijsse-Klasen et al., 2011; Tsai et al., 2011, 2020; Silaghi et al., 2012; Troyo et al., 2012; Flores-Mendoza et al., 2013; Hii et al., 2013; Jiang et al., 2013; Nogueras et al., 2013; Ramírez-Hernández et al., 2013; Giudice et al., 2014; Horta et al., 2014; Khrouf et al., 2014; Leulmi et al., 2014; Zhang et al., 2014; Peniche-Lara et al., 2015; New records of bacteria in different species of fleas from France and Spain, 2021) |
|  | *Ctenocephalides canis* | Colombia, Netherlands, **Democratic Republic of Congo**, Ivory Coast, Kenya, Laos, United Republic of Tanzania, Uruguay, UK | (Venzal et al., 2006; Berrelha et al., 2009; Varagnol et al., 2009; Tijsse-Klasen et al., 2011; Mediannikov et al., 2012b; Jiang et al., 2013; Ramírez-Hernández et al., 2013; Leulmi et al., 2014) |
|  | *Ctenocephalides orientis* | Laos | (Varagnol et al., 2009) |
|  | *Ctenophthalmus congeneroides* | Korea | (Ko et al., 2011) |
|  | *Stenoponia sidimi* | Korea | (Ko et al., 2011) |
|  | *Rhadinopsylla insolita* | Korea | (Ko et al., 2011) |
|  | *Ctenophthalmus agyrtes* | Slovakia, Lithuania | (Špitalská et al., 2015; Radzijevskaja et al., 2018) |
|  | *Ctenophthalmus solutus* | Slovakia | (Špitalská et al., 2015) |
|  | *Ctenophthalmus uncinatus* | Slovakia | (Špitalská et al., 2015) |
|  | *Ctenophthalmus calceatus* | United Republic of Tanzania | (Leulmi et al., 2014) |
|  | *Ctenophthalmus baeticus* | Spain | (New records of bacteria in different species of fleas from France and Spain, 2021) |
|  | *Ctenophtalmus sp.* | Portugal | (Stevenson et al., 2005) |
|  | *Nosopsyllus fasciatus* | Slovakia | (Špitalská et al., 2015) |
|  | *Polygenis odiosus* | Mexico | (Peniche-Lara et al., 2015) |
|  | *Polygenis axius* | Argentina | (Melis et al., 2020) |
|  | *Xenopsylla cheopis* | Algeria, Cyprus, Indonesia, Kenya, Reunion Island, USA | (Bitam et al., 2009; Karpathy et al., 2009; Barbara et al., 2010; Christou et al., 2010; Abramowicz et al., 2011; Eremeeva et al., 2012; Jiang et al., 2013; Dieme et al., 2015; Bai et al., 2017) |
|  | *Xenopsylla brasiliensis* | Reunion Island, **Democratic Republic of Congo** | (Sackal et al., 2008; Dieme et al., 2015) |
|  | *Leptopsylla segnis* | Algeria, USA | (Bitam et al., 2009; Karpathy et al., 2009) |
|  | *Pulex irritans* | Colombia, Ethiopia, Kenya, USA, Democratic Republic of Congo | (Sackal et al., 2008; Karpathy et al., 2009; Eremeeva et al., 2012; Mediannikov et al., 2012a; Jiang et al., 2013; Ramírez-Hernández et al., 2013) |
|  | *Echidnophaga gallinacea* | Kenya, USA, **Democratic Republic of Congo** | (Sackal et al., 2008; Karpathy et al., 2009; Eremeeva et al., 2012; Jiang et al., 2013; Leulmi et al., 2014) |
|  | *Archeopsylla erinacei* | Algeria, France, Spain, Portugal | (Stevenson et al., 2005; Khaldi et al., 2012; Marié et al., 2012b, 2012a; New records of bacteria in different species of fleas from France and Spain, 2021) |
|  | *Diamanus montanus* | USA | (Eremeeva et al., 2012) |
|  | *Leptopsylla aethiopica* | **Democratic Republic of Congo** | (Leulmi et al., 2014) |
|  | *Stivalius aporus* | Taiwan | (Kuo et al., 2012) |
|  | *Acropsylla episema* | Taiwan | (Kuo et al., 2012) |
|  | *Tunga penetrans* | **Democratic Republic of Congo** | (Sackal et al., 2008) |
|  | *Hystrichopsylla talpae* | Lithuania | (Radzijevskaja et al., 2018) |
|  | *Dinopsyllus lypusus* | Uganda | (Bai et al., 2017) |
|  | *Anomiopsyllus nudata* | USA | (Stevenson et al., 2005) |
| **Mosquitoes** | *Anopheles sinensis* | China | (Zhang et al., 2014, 2019) |
|  | *Culex pipiens* | China | (Zhang et al., 2014) |
|  | *Anopheles gambiae* | Côte d’Ivoire | (Socolovschi et al., 2012a) |
|  | *Anopheles arabiensis* | Senegal | (Mediannikov et al., 2013) |
|  | *Anopheles ziemanni* | Senegal | (Mediannikov et al., 2013) |
|  | *Anopheles pharoensis* | Senegal | (Mediannikov et al., 2013) |
|  | *Anopheles funestus* | Senegal | (Mediannikov et al., 2013) |
|  | *Anopheles punctipennis* | USA | (Barua et al., 2020) |
|  | *Aedes albopictus* | Gabon, China | (Zhang et al., 2019) |
|  | *Aedes luteocephalus* | Senegal | (Mediannikov et al., 2013) |
|  | *Aedes vexans* | USA | (Barua et al., 2020) |
|  | *Mansonia uniformis* | Senegal | (Mediannikov et al., 2013) |
|  | *Uranotaenia sapphirina* | USA | (Barua et al., 2020) |
|  | *Armigeres subalbatus* | China | (Zhang et al., 2019) |
| **Hard ticks** | *Rhipicephalus sanguineus* | Chile, China, Philippines, Brazil | (Abarca et al., 2013; Fang et al., 2015; de Oliveira et al., 2020) |
|  | *Rhipicephalus bursa* | Turkey | (Gargili et al., 2012) |
|  | *Rhipicephalus microplus* | Brazil | (Durães et al., 2021) |
|  | *Rhipicephalus turanicus* | Italy | (Raele et al., 2017) |
|  | *Amblyomma humerale* | Brazil | (Soares et al., 2015) |
|  | *Amblyomma maculatum* | USA | (Jiang et al., 2012) |
|  | *Amblyommma cajennense* | Brazil | (Cardoso et al., 2006) |
|  | *Dermacentor nitens* | Cuba, Brazil | (de Oliveira et al., 2020) |
|  | *Ixodes ricinus* | Romania, France | (Lejal et al., 2019; Borşan et al., 2021) |
|  | *Ixodes hexagonus* | Italy | (Pascucci et al., 2019) |
|  | *Ixodes ovatus* | Japan | (Ishikura et al., 2003) |
|  | *Dermacentor variablis* | USA | (Stanley and Rhodes, 2021) |
|  | *Haemaphysalis bancrofti* | Australia | (Chalada et al., 2018) |
|  | *Haemophysalis flava* | Japan | (Ishikura et al., 2003) |
|  | *Haemophysalis kitaokai* | Japan | (Ishikura et al., 2003) |
| **Bed bugs** | *Cimex hemipterus* | Senegal | (Mediannikov et al., 2013) |
| **Mites** | *Eulaelaps stabularis* | China | (Fang et al., 2015) |
|  | *Hyperlaelaps microti* | Lithuania | (Radzijevskaja et al., 2018) |
|  | *Laelaps agilis* | Lithuania | (Radzijevskaja et al., 2018) |
|  | *Trombiculid* | South Korea | (Choi et al., 2007) |
|  | *Leptotrombidium deliense* | Taiwan | (Tsui et al., 2007) |
|  | *Mesostigmata* | Taiwan | (Tsui et al., 2007) |
| **Lice** | *Liposcelis bostrychophila* | Australia, USA, Canada | (Behar et al., 2010; Thepparit et al., 2011) |
|  | *Linognathus setosus* | China | (Zhang et al., 2014) |
|  | *Pediculus humanus humanus* | **Democratic Republic of Congo** | This study |
|  | *Pediculus humanus capitis* | **Democratic Republic of Congo** | This study |

**References**

Abarca, K., López, J., Acosta-Jamett, G., and Martínez-Valdebenito, C. (2013). Rickettsia felis in Rhipicephalus sanguineus from two distant Chilean cities. *Vector Borne Zoonotic Dis. Larchmt. N* 13, 607–609. doi:10.1089/vbz.2012.1201.

Abramowicz, K. F., Rood, M. P., Krueger, L., and Eremeeva, M. E. (2011). Urban focus of Rickettsia typhi and Rickettsia felis in Los Angeles, California. *Vector Borne Zoonotic Dis. Larchmt. N* 11, 979–984. doi:10.1089/vbz.2010.0117.

Amanzougaghene, N., Fenollar, F., Sangaré, A. K., Sissoko, M. S., Doumbo, O. K., Raoult, D., et al. (2017). Detection of bacterial pathogens including potential new species in human head lice from Mali. *PLOS ONE* 12, e0184621. doi:10.1371/journal.pone.0184621.

Angelakis, E., Diatta, G., Abdissa, A., Trape, J.-F., Mediannikov, O., Richet, H., et al. (2011). Altitude-dependent Bartonella quintana genotype C in head lice, Ethiopia. *Emerg. Infect. Dis.* 17, 2357–2359. doi:10.3201/eid1712.110453.

Bai, Y., Osikowicz, L. M., Kosoy, M. Y., Eisen, R. J., Atiku, L. A., Mpanga, J. T., et al. (2017). Comparison of Zoonotic Bacterial Agents in Fleas Collected from Small Mammals or Host-Seeking Fleas from a Ugandan Region Where Plague Is Endemic. *mSphere* 2, e00402-17. doi:10.1128/mSphere.00402-17.

Barbara, K. A., Farzeli, A., Ibrahim, I. N., Antonjaya, U., Yunianto, A., Winoto, I., et al. (2010). Rickettsial infections of fleas collected from small mammals on four islands in Indonesia. *J. Med. Entomol.* 47, 1173–1178. doi:10.1603/me10064.

Barua, S., Hoque, M. M., Kelly, P. J., Poudel, A., Adekanmbi, F., Kalalah, A., et al. (2020). First report of Rickettsia felis in mosquitoes, USA. *Emerg. Microbes Infect.* 9, 1008–1010. doi:10.1080/22221751.2020.1760736.

Behar, A., McCormick, L. J., and Perlman, S. J. (2010). Rickettsia felis infection in a common household insect pest, Liposcelis bostrychophila (Psocoptera: Liposcelidae). *Appl. Environ. Microbiol.* 76, 2280–2285. doi:10.1128/AEM.00026-10.

Bermúdez, C. S. E., Zaldívar, A. Y., Spolidorio, M. G., Moraes-Filho, J., Miranda, R. J., Caballero, C. M., et al. (2011). Rickettsial infection in domestic mammals and their ectoparasites in El Valle de Antón, Coclé, Panamá. *Vet. Parasitol.* 177, 134–138. doi:10.1016/j.vetpar.2010.11.020.

Berrelha, J., Briolant, S., Muller, F., Rolain, J.-M., Marie, J.-L., Pagés, F., et al. (2009). Rickettsia felis and Rickettsia massiliae in Ivory Coast, Africa. *Clin. Microbiol. Infect. Off. Publ. Eur. Soc. Clin. Microbiol. Infect. Dis.* 15 Suppl 2, 251–252. doi:10.1111/j.1469-0691.2008.02273.x.

Bitam, I., Baziz, B., Kernif, T., Harrat, Z., Parola, P., and Raoult, D. (2009). Molecular detection of Rickettsia typhi and Rickettsia felis in fleas from Algeria. *Clin. Microbiol. Infect.* 15, 255–256. doi:10.1111/j.1469-0691.2008.02275.x.

Borşan, S.-D., Ionică, A. M., Galon, C., Toma-Naic, A., Peştean, C., Sándor, A. D., et al. (2021). High Diversity, Prevalence, and Co-infection Rates of Tick-Borne Pathogens in Ticks and Wildlife Hosts in an Urban Area in Romania. *Front. Microbiol.* 12, 645002. doi:10.3389/fmicb.2021.645002.

Bouvresse, S., Socolovshi, C., Berdjane, Z., Durand, R., Izri, A., Raoult, D., et al. (2011). No evidence of Bartonella quintana but detection of Acinetobacter baumannii in head lice from elementary schoolchildren in Paris. *Comp. Immunol. Microbiol. Infect. Dis.* 34, 475–477. doi:10.1016/j.cimid.2011.08.007.

Cardoso, L. D., Freitas, R. N., Mafra, C. L., Neves, C. V. B., Figueira, F. C. B., Labruna, M. B., et al. (2006). [Characterization of Rickettsia spp. circulating in a silent peri-urban focus for Brazilian spotted fever in Caratinga, Minas Gerais, Brazil]. *Cad. Saude Publica* 22, 495–501. doi:10.1590/s0102-311x2006000300004.

Chalada, M. J., Stenos, J., Vincent, G., Barker, D., and Bradbury, R. S. (2018). A Molecular Survey of Tick-Borne Pathogens from Ticks Collected in Central Queensland, Australia. *Vector Borne Zoonotic Dis. Larchmt. N* 18, 151–163. doi:10.1089/vbz.2017.2182.

Choi, Y.-J., Lee, E.-M., Park, J.-M., Lee, K.-M., Han, S.-H., Kim, J.-K., et al. (2007). Molecular detection of various rickettsiae in mites (acari: trombiculidae) in southern Jeolla Province, Korea. *Microbiol. Immunol.* 51, 307–312. doi:10.1111/j.1348-0421.2007.tb03912.x.

Christou, C., Psaroulaki, A., Antoniou, M., Toumazos, P., Ioannou, I., Mazeris, A., et al. (2010). Rickettsia typhi and Rickettsia felis in Xenopsylla cheopis and Leptopsylla segnis Parasitizing Rats in Cyprus. *Am. J. Trop. Med. Hyg.* 83, 1301–1304. doi:10.4269/ajtmh.2010.10-0118.

Dahmana, H., Amanzougaghene, N., Davoust, B., Normand, T., Carette, O., Demoncheaux, J.-P., et al. (2019). Great diversity of Piroplasmida in Equidae in Africa and Europe, including potential new species. *Vet. Parasitol. Reg. Stud. Rep.* 18, 100332. doi:10.1016/j.vprsr.2019.100332.

Dahmani, M., Davoust, B., Benterki, M. S., Fenollar, F., Raoult, D., and Mediannikov, O. (2015). Development of a new PCR-based assay to detect Anaplasmataceae and the first report of Anaplasma phagocytophilum and Anaplasma platys in cattle from Algeria. *Comp. Immunol. Microbiol. Infect. Dis.* 39, 39–45. doi:10.1016/j.cimid.2015.02.002.

de Oliveira, J. C. P., Reckziegel, G. H., Ramos, C. A. do N., Giannelli, A., Alves, L. C., de Carvalho, G. A., et al. (2020). Detection of Rickettsia felis in ectoparasites collected from domestic animals. *Exp. Appl. Acarol.* 81, 255–264. doi:10.1007/s10493-020-00505-2.

Dieme, C., Parola, P., Guernier, V., Lagadec, E., Le Minter, G., Balleydier, E., et al. (2015). Rickettsia and Bartonella Species in Fleas from Reunion Island. *Am. J. Trop. Med. Hyg.* 92, 617–619. doi:10.4269/ajtmh.14-0424.

Durães, L. S., Bitencourth, K., Ramalho, F. R., Nogueira, M. C., Nunes, E. de C., and Gazêta, G. S. (2021). Biodiversity of Potential Vectors of Rickettsiae and Epidemiological Mosaic of Spotted Fever in the State of Paraná, Brazil. *Front. Public Health* 9, 577789. doi:10.3389/fpubh.2021.577789.

Eremeeva, M. E., Karpathy, S. E., Krueger, L., Hayes, E. K., Williams, A. M., Zaldivar, Y., et al. (2012). Two pathogens and one disease: detection and identification of flea-borne Rickettsiae in areas endemic for murine typhus in California. *J. Med. Entomol.* 49, 1485–1494. doi:10.1603/me11291.

Fang, W., Yao, L., Cao, X., Sun, Y., Zhang, X., Shao, L., et al. (2015). First Molecular Detection of Rickettsia felis-Like Organism in Eulaelaps stabularis from the Changbai Mountain Area of China. *J. Parasitol.* 101, 514–519. doi:10.1645/14-695.1.

Flores-Mendoza, C., Florin, D., Felices, V., Pozo, E. J., Graf, P. C. F., Burrus, R. G., et al. (2013). Detection of Rickettsia parkeri from within Piura, Peru, and the first reported presence of Candidatus Rickettsia andeanae in the tick Rhipicephalus sanguineus. *Vector Borne Zoonotic Dis. Larchmt. N* 13, 505–508. doi:10.1089/vbz.2012.1028.

Gargili, A., Palomar, A. M., Midilli, K., Portillo, A., Kar, S., and Oteo, J. A. (2012). Rickettsia Species in Ticks Removed from Humans in Istanbul, Turkey. *Vector Borne Zoonotic Dis.* 12, 938–941. doi:10.1089/vbz.2012.0996.

Gehrke, F. S., Gazeta, G. S., Souza, E. R., Ribeiro, A., Marrelli, M. T., and Schumaker, T. T. S. (2009). Rickettsia rickettsii, Rickettsia felis and Rickettsia sp. TwKM03 infecting Rhipicephalus sanguineus and Ctenocephalides felis collected from dogs in a Brazilian spotted fever focus in the State of Rio De Janeiro/Brazil. *Clin. Microbiol. Infect. Off. Publ. Eur. Soc. Clin. Microbiol. Infect. Dis.* 15 Suppl 2, 267–268. doi:10.1111/j.1469-0691.2008.02229.x.

Giudice, E., Di Pietro, S., Alaimo, A., Blanda, V., Lelli, R., Francaviglia, F., et al. (2014). A molecular survey of Rickettsia felis in fleas from cats and dogs in Sicily (Southern Italy). *PloS One* 9, e106820. doi:10.1371/journal.pone.0106820.

Hii, S.-F., Abdad, M. Y., Kopp, S. R., Stenos, J., Rees, R. L., and Traub, R. J. (2013). Seroprevalence and risk factors for Rickettsia felis exposure in dogs from Southeast Queensland and the Northern Territory, Australia. *Parasit. Vectors* 6, 159. doi:10.1186/1756-3305-6-159.

Hornok, S., Meli, M. L., Perreten, A., Farkas, R., Willi, B., Beugnet, F., et al. (2010). Molecular investigation of hard ticks (Acari: Ixodidae) and fleas (Siphonaptera: Pulicidae) as potential vectors of rickettsial and mycoplasmal agents. *Vet. Microbiol.* 140, 98–104. doi:10.1016/j.vetmic.2009.07.013.

Horta, M. C., Ogrzewalska, M., Azevedo, M. C., Costa, F. B., Ferreira, F., and Labruna, M. B. (2014). Rickettsia felis in Ctenocephalides felis felis from five geographic regions of Brazil. *Am. J. Trop. Med. Hyg.* 91, 96–100. doi:10.4269/ajtmh.13-0699.

Hun, L., Troyo, A., Taylor, L., Barbieri, A. M., and Labruna, M. B. (2011). First report of the isolation and molecular characterization of Rickettsia amblyommii and Rickettsia felis in Central America. *Vector Borne Zoonotic Dis. Larchmt. N* 11, 1395–1397. doi:10.1089/vbz.2011.0641.

Ishikura, M., Ando, S., Shinagawa, Y., Matsuura, K., Hasegawa, S., Nakayama, T., et al. (2003). Phylogenetic analysis of spotted fever group rickettsiae based on gltA, 17-kDa, and rOmpA genes amplified by nested PCR from ticks in Japan. *Microbiol. Immunol.* 47, 823–832. doi:10.1111/j.1348-0421.2003.tb03448.x.

Jiang, J., Maina, A. N., Knobel, D. L., Cleaveland, S., Laudisoit, A., Wamburu, K., et al. (2013). Molecular detection of Rickettsia felis and Candidatus Rickettsia asemboensis in fleas from human habitats, Asembo, Kenya. *Vector Borne Zoonotic Dis. Larchmt. N* 13, 550–558. doi:10.1089/vbz.2012.1123.

Jiang, J., Stromdahl, E. Y., and Richards, A. L. (2012). Detection of Rickettsia parkeri and Candidatus Rickettsia andeanae in Amblyomma maculatum Gulf Coast ticks collected from humans in the United States. *Vector Borne Zoonotic Dis. Larchmt. N* 12, 175–182. doi:10.1089/vbz.2011.0614.

Karpathy, S. E., Hayes, E. K., Williams, A. M., Hu, R., Krueger, L., Bennett, S., et al. (2009). Detection of Rickettsia felis and Rickettsia typhi in an area of California endemic for murine typhus. *Clin. Microbiol. Infect. Off. Publ. Eur. Soc. Clin. Microbiol. Infect. Dis.* 15 Suppl 2, 218–219. doi:10.1111/j.1469-0691.2008.02140.x.

Kelly, P. J., Lucas, H., Eremeeva, M. E., Dirks, K. G., Rolain, J. M., Yowell, C., et al. (2010). Rickettsia felis, West Indies. *Emerg. Infect. Dis.* 16, 570–571. doi:10.3201/eid1603.091431.

Khaldi, M., Socolovschi, C., Benyettou, M., Barech, G., Biche, M., Kernif, T., et al. (2012). Rickettsiae in arthropods collected from the North African Hedgehog (Atelerix algirus) and the desert hedgehog (Paraechinus aethiopicus) in Algeria. *Comp. Immunol. Microbiol. Infect. Dis.* 35, 117–122. doi:10.1016/j.cimid.2011.11.007.

Khrouf, F., M’Ghirbi, Y., Znazen, A., Ben Jemaa, M., Hammami, A., and Bouattour, A. (2014). Detection of Rickettsia in Rhipicephalus sanguineus ticks and Ctenocephalides felis fleas from southeastern Tunisia by reverse line blot assay. *J. Clin. Microbiol.* 52, 268–274. doi:10.1128/JCM.01925-13.

Ko, S., Kim, H.-C., Yang, Y.-C., Chong, S.-T., Richards, A. L., Sames, W. J., et al. (2011). Detection of Rickettsia felis and Rickettsia typhi and seasonal prevalence of fleas collected from small mammals at Gyeonggi Province in the Republic of Korea. *Vector Borne Zoonotic Dis. Larchmt. N* 11, 1243–1251. doi:10.1089/vbz.2010.0261.

Kuo, C. C., Huang, J. L., Lin, T. E., and Wang, H. C. (2012). Detection of Rickettsia spp. and host and habitat associations of fleas (Siphonaptera) in eastern Taiwan. *Med. Vet. Entomol.* 26, 341–350. doi:10.1111/j.1365-2915.2012.01009.x.

La Scola, B., Gundi, V. A. K. B., Khamis, A., and Raoult, D. (2006). Sequencing of the rpoB gene and flanking spacers for molecular identification of Acinetobacter species. *J. Clin. Microbiol.* 44, 827–832. doi:10.1128/JCM.44.3.827-832.2006.

Lejal, E., Moutailler, S., Šimo, L., Vayssier-Taussat, M., and Pollet, T. (2019). Tick-borne pathogen detection in midgut and salivary glands of adult Ixodes ricinus. *Parasit. Vectors* 12, 152. doi:10.1186/s13071-019-3418-7.

Leulmi, H., Socolovschi, C., Laudisoit, A., Houemenou, G., Davoust, B., Bitam, I., et al. (2014). Detection of Rickettsia felis, Rickettsia typhi, Bartonella Species and Yersinia pestis in Fleas (Siphonaptera) from Africa. *PLoS Negl. Trop. Dis.* 8, e3152. doi:10.1371/journal.pntd.0003152.

Li, W., Ortiz, G., Fournier, P.-E., Gimenez, G., Reed, D. L., Pittendrigh, B., et al. (2010). Genotyping of human lice suggests multiple emergencies of body lice from local head louse populations. *PLoS Negl. Trop. Dis.* 4, e641. doi:10.1371/journal.pntd.0000641.

Marié, J.-L., Davoust, B., Socolovschi, C., Mediannikov, O., Roqueplo, C., Beaucournu, J.-C., et al. (2012a). Rickettsiae in arthropods collected from red foxes (Vulpes vulpes) in France. *Comp. Immunol. Microbiol. Infect. Dis.* 35, 59–62. doi:10.1016/j.cimid.2011.10.001.

Marié, J.-L., Davoust, B., Socolovschi, C., Raoult, D., and Parola, P. (2012b). Molecular detection of rickettsial agents in ticks and fleas collected from a European hedgehog (Erinaceus europaeus) in Marseilles, France. *Comp. Immunol. Microbiol. Infect. Dis.* 35, 77–79. doi:10.1016/j.cimid.2011.11.005.

Mba, P. A., Marié, J.-L., Rolain, J.-M., Davoust, B., Beaucournu, J.-C., Raoult, D., et al. (2011). Rickettsia felis and Bartonella henselae in fleas from Lebanon. *Vector Borne Zoonotic Dis. Larchmt. N* 11, 991–992. doi:10.1089/vbz.2010.0146.

Mediannikov, O., Abdissa, A., Diatta, G., Trape, J.-F., and Raoult, D. (2012a). Rickettsia felis in fleas, southern Ethiopia, 2010. *Emerg. Infect. Dis.* 18, 1385–1386. doi:10.3201/eid1808.111243.

Mediannikov, O., Cabre, O., Qu, F., Socolovschi, C., Davoust, B., Marié, J.-L., et al. (2011). Rickettsia felis and Bartonella clarridgeiae in fleas from New Caledonia. *Vector Borne Zoonotic Dis. Larchmt. N* 11, 181–183. doi:10.1089/vbz.2009.0199.

Mediannikov, O., Davoust, B., Socolovschi, C., Tshilolo, L., Raoult, D., and Parola, P. (2012b). Spotted fever group rickettsiae in ticks and fleas from the Democratic Republic of the Congo. *Ticks Tick-Borne Dis.* 3, 371–373. doi:10.1016/j.ttbdis.2012.10.015.

Mediannikov, O., Fenollar, F., Socolovschi, C., Diatta, G., Bassene, H., Molez, J.-F., et al. (2010). Coxiella burnetii in humans and ticks in rural Senegal. *PLoS Negl. Trop. Dis.* 4, e654. doi:10.1371/journal.pntd.0000654.

Mediannikov, O., Socolovschi, C., Edouard, S., Fenollar, F., Mouffok, N., Bassene, H., et al. (2013). Common Epidemiology of Rickettsia felis Infection and Malaria, Africa. *Emerg. Infect. Dis.* 19, 1775–1783. doi:10.3201/eid1911.130361.

Melis, M., Espinoza-Carniglia, M., Savchenko, E., Nava, S., and Lareschi, M. (2020). Molecular detection and identification of Rickettsia felis in Polygenis (Siphonaptera, Rhopalopsyllidae, Rhopalopsyllinae) associated with cricetid rodents in a rural area from central Argentina. *Vet. Parasitol. Reg. Stud. Rep.* 21, 100445. doi:10.1016/j.vprsr.2020.100445.

New records of bacteria in different species of fleas from France and Spain (2021). *Comp. Immunol. Microbiol. Infect. Dis.* 76, 101648. doi:10.1016/j.cimid.2021.101648.

Nguyen-Hieu, T., Aboudharam, G., Signoli, M., Rigeade, C., Drancourt, M., and Raoult, D. (2010). Evidence of a louse-borne outbreak involving typhus in Douai, 1710-1712 during the war of Spanish succession. *PloS One* 5, e15405. doi:10.1371/journal.pone.0015405.

Nogueras, M. M., Pons, I., Ortuño, A., Miret, J., Pla, J., Castellà, J., et al. (2013). Molecular detection of Rickettsia typhi in cats and fleas. *PloS One* 8, e71386. doi:10.1371/journal.pone.0071386.

Parola, P., Diatta, G., Socolovschi, C., Mediannikov, O., Tall, A., Bassene, H., et al. (2011). Tick-borne relapsing fever borreliosis, rural senegal. *Emerg. Infect. Dis.* 17, 883–885. doi:10.3201/eid1705.100573.

Pascucci, I., Di Domenico, M., Curini, V., Cocco, A., Averaimo, D., D’Alterio, N., et al. (2019). Diversity of Rickettsia in Ticks Collected in Abruzzi and Molise Regions (Central Italy). *Microorganisms* 7, 696. doi:10.3390/microorganisms7120696.

Peniche-Lara, G., Dzul-Rosado, K., Pérez-Osorio, C., and Zavala-Castro, J. (2015). Rickettsia typhi in rodents and R. felis in fleas in Yucatán as a possible causal agent of undefined febrile cases. *Rev. Inst. Med. Trop. Sao Paulo* 57, 129–132. doi:10.1590/S0036-46652015000200005.

Radzijevskaja, J., Kaminskienė, E., Lipatova, I., Mardosaitė-Busaitienė, D., Balčiauskas, L., Stanko, M., et al. (2018). Prevalence and diversity of Rickettsia species in ectoparasites collected from small rodents in Lithuania. *Parasit. Vectors* 11, 375. doi:10.1186/s13071-018-2947-9.

Raele, D. A., Galante, D., Pugliese, N., Salandra, G. L., and Cafiero, M. A. (2017). Spotted fever group rickettsiae associated with ixodid ticks in wild environment in Southern Italy. *MicrobiologyOpen* 7, e00527. doi:10.1002/mbo3.527.

Ramírez-Hernández, A., Montoya, V., Martínez, A., Pérez, J. E., Mercado, M., de la Ossa, A., et al. (2013). Molecular detection of Rickettsia felis in different flea species from Caldas, Colombia. *Am. J. Trop. Med. Hyg.* 89, 453–459. doi:10.4269/ajtmh.12-0698.

Rolain, J.-M., Franc, M., Davoust, B., and Raoult, D. (2003). Molecular Detection of Bartonella quintana, B. koehlerae, B. henselae, B. clarridgeiae, Rickettsia felis, and Wolbachia pipientis in Cat Fleas, France. *Emerg. Infect. Dis.* 9, 339–342. doi:10.3201/eid0903.020278.

Sackal, C., Laudisoit, A., Kosoy, M., Massung, R., Eremeeva, M. E., Karpathy, S. E., et al. (2008). Bartonella spp. and Rickettsia felis in Fleas, Democratic Republic of Congo. *Emerg. Infect. Dis.* 14, 1972–1974. doi:10.3201/eid1412.080610.

Silaghi, C., Knaus, M., Rapti, D., Shukullari, E., Pfister, K., and Rehbein, S. (2012). Rickettsia felis and Bartonella spp. in fleas from cats in Albania. *Vector Borne Zoonotic Dis. Larchmt. N* 12, 76–77. doi:10.1089/vbz.2011.0732.

Soares, H. S., Barbieri, A. R. M., Martins, T. F., Minervino, A. H. H., de Lima, J. T. R., Marcili, A., et al. (2015). Ticks and rickettsial infection in the wildlife of two regions of the Brazilian Amazon. *Exp. Appl. Acarol.* 65, 125–140. doi:10.1007/s10493-014-9851-6.

Socolovschi, C., Mediannikov, O., Sokhna, C., Tall, A., Diatta, G., Bassene, H., et al. (2010). Rickettsia felis-associated uneruptive fever, Senegal. *Emerg. Infect. Dis.* 16, 1140–1142. doi:10.3201/eid1607.100070.

Socolovschi, C., Pages, F., Ndiath, M. O., Ratmanov, P., and Raoult, D. (2012a). Rickettsia Species in African Anopheles Mosquitoes. *PLoS ONE* 7, e48254. doi:10.1371/journal.pone.0048254.

Socolovschi, C., Pagés, F., and Raoult, D. (2012b). Rickettsia felis in Aedes albopictus mosquitoes, Libreville, Gabon. *Emerg. Infect. Dis.* 18, 1687–1689. doi:10.3201/eid1810.120178.

Sokhna, C., Mediannikov, O., Fenollar, F., Bassene, H., Diatta, G., Tall, A., et al. (2013). Point-of-Care Laboratory of Pathogen Diagnosis in Rural Senegal. *PLoS Negl. Trop. Dis.* 7, e1999. doi:10.1371/journal.pntd.0001999.

Špitalská, E., Boldiš, V., Mošanský, L., Sparagano, O., and Stanko, M. (2015). Rickettsia species in fleas collected from small mammals in Slovakia. *Parasitol. Res.* 114, 4333–4339. doi:10.1007/s00436-015-4713-7.

Stanley, H., and Rhodes, D. V. L. (2021). Presence of Rickettsia Species in Ticks Collected from Companion Animals in Northeastern Georgia, United States. *Vet. Sci.* 8, 37. doi:10.3390/vetsci8030037.

Stevenson, H. L., Labruna, M. B., Montenieri, J. A., Kosoy, M. Y., Gage, K. L., and Walker, D. H. (2005). Detection of Rickettsia felis in a New World flea species, Anomiopsyllus nudata (Siphonaptera: Ctenophthalmidae). *J. Med. Entomol.* 42, 163–167. doi:10.1093/jmedent/42.2.163.

Thepparit, C., Sunyakumthorn, P., Guillotte, M. L., Popov, V. L., Foil, L. D., and Macaluso, K. R. (2011). Isolation of a rickettsial pathogen from a non-hematophagous arthropod. *PloS One* 6, e16396. doi:10.1371/journal.pone.0016396.

Tijsse-Klasen, E., Fonville, M., Gassner, F., Nijhof, A. M., Hovius, E. K. E., Jongejan, F., et al. (2011). Absence of zoonotic Bartonella species in questing ticks: first detection of Bartonella clarridgeiae and Rickettsia felis in cat fleas in the Netherlands. *Parasit. Vectors* 4, 61. doi:10.1186/1756-3305-4-61.

Troyo, A., Álvarez, D., Taylor, L., Abdalla, G., Calderón-Arguedas, Ó., Zambrano, M. L., et al. (2012). Rickettsia felis in Ctenocephalides felis from Guatemala and Costa Rica. *Am. J. Trop. Med. Hyg.* 86, 1054–1056. doi:10.4269/ajtmh.2012.11-0742.

Tsai, K.-H., Huang, C.-G., Fang, C.-T., Shu, P.-Y., Huang, J.-H., and Wu, W.-J. (2011). Prevalence of Rickettsia felis and the first identification of Bartonella henselae Fizz/CAL-1 in cat fleas (Siphonaptera: Pulicidae) from Taiwan. *J. Med. Entomol.* 48, 445–452. doi:10.1603/me10022.

Tsai, K.-H., Yen, T.-Y., Wu, W.-J., Carvalho, R., Raoult, D., and Fournier, P.-E. (2020). Investigation of Ctenocephalides felis on domestic dogs and Rickettsia felis infection in the Democratic Republic of Sao Tome and Principe. *Zoonoses Public Health* 67, 892–902. doi:10.1111/zph.12776.

Tsui, P.-Y., Tsai, K.-H., Weng, M.-H., Hung, Y.-W., Liu, Y.-T., Hu, K.-Y., et al. (2007). Molecular detection and characterization of spotted fever group rickettsiae in Taiwan. *Am. J. Trop. Med. Hyg.* 77, 883–890.

Varagnol, M., Parola, P., Jouan, R., Beaucournu, J.-C., Rolain, J.-M., and Raoult, D. (2009). First detection of Rickettsia felis and Bartonella clarridgeiae in fleas from Laos. *Clin. Microbiol. Infect. Off. Publ. Eur. Soc. Clin. Microbiol. Infect. Dis.* 15 Suppl 2, 334–335. doi:10.1111/j.1469-0691.2008.02272.x.

Venzal, J. M., Pérez-Martínez, L., Félix, M. L., Portillo, A., Blanco, J. R., and Oteo, J. A. (2006). Prevalence of Rickettsia felis in Ctenocephalides felis and Ctenocephalides canis from Uruguay. *Ann. N. Y. Acad. Sci.* 1078, 305–308. doi:10.1196/annals.1374.056.

Zhang, J., Lu, G., Kelly, P., Zhang, Z., Wei, L., Yu, D., et al. (2014). First report of Rickettsia felis in China. *BMC Infect. Dis.* 14, 682. doi:10.1186/s12879-014-0682-1.

Zhang, J., Lu, G., Li, J., Kelly, P., Li, M., Wang, J., et al. (2019). Molecular Detection of Rickettsia felis and Rickettsia bellii in Mosquitoes. *Vector Borne Zoonotic Dis. Larchmt. N* 19, 802–809. doi:10.1089/vbz.2019.2456.
